# New Metrics for Cross-Country Comparison of Scientific Impact

**DOI:** 10.3389/frma.2020.594891

**Published:** 2020-10-30

**Authors:** Renan Moritz V. R. Almeida, Luis Fabiano F. Borges, Daniel C. Moreira, Marcelo Hermes-Lima

**Affiliations:** ^1^ Programa de Engenharia Biomédica, Coordenação dos Programas de Pós-Graduação em Engenharia (COPPE), Universidade Federal do Rio de Janeiro, Rio de Janeiro, Brazil; ^2^ Coordenação de Aperfeiçoamento de Pessoal de Nível Superior, Ministério da Educação, Brasilia, Brazil; ^3^ Faculdade de Medicina, Universidade de Brasília, Brasilia, Brazil; ^4^ Departamento de Biologia Celular, Universidade de Brasília, Brasilia, Brazil

**Keywords:** citations per publication, h-index, scientific productivity, performance metrics, science impact

## Introduction

Comparing scientific impact among countries is fundamental for the development of effective strategies for science financing and assessment (Vinkler, 1986; King, 2004; Hermes-Lima et al., 2007a; Wagner et al., 2018). Such comparisons allow the definition of funding strategies based on inputs and outputs of the Research and Development (R&D) system and the identification of countries and institutions with the best performance (Freeman, 1977; Leydesdorff et al., 2019). These top performer countries, in turn, can be emulated by less-developed nations.

Usually, the number of scientific papers and the number of citations of a country are the main variables used for these comparisons. These metrics are frequently combined in a single indicator, such as the Citations Per Publication (CPP) (Lehmann et al., 2003; Moed, 2005; Waltman and van Eck, 2009; Vinkler, 2010) or the well-known h-index (Hirsch, 2005; Egghe, 2006; Glänzel, 2006; Hermes-Lima et al., 2007b; Franceschini and Maisano, 2010). However, each index has its caveats, and identifying simple, meaningful metrics for scientific productivity evaluation at country level has been a challenge (King, 2004; Leydesdorff et al., 2019). The limitations of the h-index have been vastly discussed in the literature (Bornmann and Daniel, 2005; Waltman and van Eck, 2012); and inter-country comparisons using the CPP have problems related to the time frame of an analysis. Thus, early publications from a given country will have more citations (therefore resulting in a higher CPP) than later publications, simply because papers published earlier had more time to be cited.

For instance, Belgium was the country with the highest research impact in Medicine in 2019 (Scimago data), with a CPP value of 1.38 (12,768 publications; 17,595 citations), whereas its CPP value for 2016 is 17.73 (11,766 publications; 208,622 citations) and for 2013, 30.91 (22 times higher than in 2019). This limitation leads to the question: How can one avoid this caveat and meaningfully compare research impact among countries in a year-per-year basis?

In this essay we present two simple methods to standardize CPP values along time, thus allowing for comparisons among countries in a time-independent fashion. Therefore, two metrics are presented: The Relative CPP and the Impact-Relative Rank Score (IRRS). Both of these procedures are based on the principle of normalizing the metric according to maximum-minimum values in the common set of observational units (countries) used for comparison.

## Metrics Definition

First, consider the CPP definition for a given country and year as the number of citations received by the country’s publications in that year, divided by the number of those publications (Vinkler, 2010). Next, in a set of selected countries, the maximum observed CPP value is defined as “100%,” and, from this maximum value, one can then calculate the percentage of the CPPs for the remaining countries: The Relative CPPs in the data set, for each country. The second procedure, the IRRS, takes the additional step of ranking countries according to their Relative CPPs, after what their rank order is used to calculate a score in a 0 to 10 scale (Equation 1). In this scale, the value “zero” is assigned to the last-ranked country and “10” to the first placed one (the “benchmark country”).

$$\text{Impact Relative Rank Scores }\left(\text{IRRS}\right)=\left[1-\left(\frac{\text{Country position}}{\text{Total number of countries}}\right)\right]\times 10$$ (1)

## Examples

In general, the inclusion of countries with a modest number of publications per year (such as small islands) leads to atypical CPP values, distorting a metric and thus reducing its efficacy. In order to minimize this effect, a cut-off value is commonly used for country selection, preferably resulting in the inclusion of more than 95% of all publications in a base year. Therefore, below, the approach is illustrated for the field of Medicine, with a cut-off of 2,000 papers/year (for the 2019 dataset, this threshold resulted in 52 countries representing 95.7% of the 1.21 million available publications).

At first, the ranking of the 52 selected countries according to their CPPs in Medicine was obtained from the Scimago platform (Scimago Lab, 2019). In this rank, Belgium was the benchmark country for 2019, with CPP = 1.38; and the Netherlands was in the second position (CPP = 1.28). Belgium was also the benchmark in 2018, 2016, and 2013; and it ranked second to fifth in 2017, 2015, 2014, 2012, and 2011. Other CPP values for countries leading the impact rankings were (Medicine): 5.94 for 2018 (Belgium), 12.40 for 2017 (Finland), 17.73 for 2016 (Belgium), 25.02 for 2015 (Finland), 27.66 for 2014 (Finland), 30.91 for 2013 (Belgium), 36.10 for 2012 (Singapore), and 38.56 for 2011 (the Netherlands).

As mentioned, taking into account the CPP value of the benchmark country, it is possible, then, to calculate the impact of other countries relatively to it in each year. For example, medical United Kingdom papers had CPP = 1.07 in 2019 and CPP = 4.71 in 2018. Therefore, according to the Relative CPP procedure described above, the relative impact of the United Kingdom in those years was 77.5 and 79.3% respectively - the United Kingdom Relative CPPs.

**FIGURE 1:**
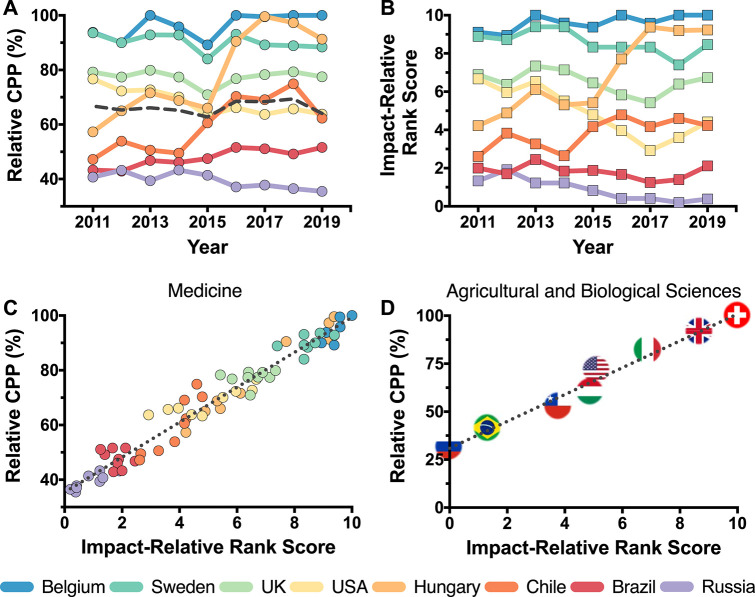
New metrics for cross-country comparison of scientific impact. **(A)** The relative citations per publication (CPP); and **(B)** The impact-relative rank score (IRSS) for medical publications, 2011–2019, Belgium, Brazil, Chile, Hungary, Russia, Sweden, the United Kingdom, the United States, and the world average (dashed lines). **(C)** Relative CPP vs. IRSS (dotted lined: linear regression *y* = 35.42 + 6.39*x*; R^2^ = 0.96; data from panels **A** and **B**). **(D)** Relative CPP vs. IRSS, agricultural and biological area. Russia, Brazil, Chile, Italy, the United States, Hungary, the United Kingdom, and Switzerland, 2018 (dotted line: linear regression *y* = 30.94 + 6.99*x*; R^2^ = 0.98). All data retrieved from Scimago (Scimago Lab, 2019) (https://www.scimagojr.com).

Figure 1A, presents the 2011–2019 Relative CPPs for the Medical area in eight countries, selected as to give a comprehensive overview of the world scientific productivity in the area: Belgium, Brazil, Chile, Hungary, Russia, Sweden, the United Kingdom, the United States and the World average (dashed line). It may be seen that the United Kingdom and Sweden remained relatively stable in the period, with 70–79% and 88–93% Relative CPPs, respectively. It is also important to highlight the rise of Hungary, from 57% in 2011 to above 90% in 2016–2019, and, in fact, the impact of medical Hungarian publications, in 2016–2017, becomes higher than those of the United Kingdom and Sweden. Similarly, Chilean impact rose from 47–54% in 2011–2014 to above 60% in the following years, and Brazilian Medical science also presented a (modest) rise, from 42–43% Relative CPPs in 2011–2012 to 49–51% in 2016–2019. Finally, differing from the above countries (all on the rise or stable), the impact of Russian and US American medical publications decreased in the period. The world Relative CPP in Medicine ranged from 62.8 to 69.4%, 2011 to 2019 (Panel **A**).

The IRRS in Medicine for the same set of countries is shown in Panel **B**. As expected, most country trends in the yearly-based IRSS scores are similar to those based on the Relative CPPs. The IRRS scores of Belgium, Sweden and the United Kingdom are clearly stable, while Chile and Hungary presented a rise in their values. The United States and Russia experienced a decrease in the period. Brazil, on the other hand, shows a reasonably stable IRSS, while, as mentioned, its Relative CPPs presented a modest rise.

The Linear Coefficient of Determination between the Relative CPPs and the IRRS values for Medicine (Panel **C**) showed a high correlation between these metrics (dotted line: linear regression *y* = 35.42 + 6.39*x*; R^2^ = 0.96; data from panels **A** and **B**). Panel **D** shows that Relative CPPs and IRSS values are also highly correlated in the Agricultural and Biological Sciences, 2018, for Russia, Brazil, Chile, Italy, the United States, Hungary, the United Kingdom, and Switzerland (dotted line: linear regression *y* = 30.94 + 6.99*x*; R^2^ = 0.98). The country with the highest impact in this area in 2018 (minimum of 1,000 publications) was Switzerland, with CPP = 4.92. Finally, Table 1 contrasts the benchmark country to the World average and to an example country (Brazil) in ten Scimago subject areas, 2018.

**TABLE 1:** Citations per publication (CPP), Relative CPP, and impact-relative rank score (IRRS) metrics for documents published in 2018, ten subject areas (www.scimagojr.com): benchmark country, world average and country example (Brazil).

Subject area	Cut-off (documents)	Countries	Benchmark Country		World		Brazil		
			CPP	Country	CPP	Relative CPP	CPP	Relative CPP	IRRS
Agricultural and Biological	1,000	45	4.92	Switzerland	3.17	64.5	2.04	41.5	1.33
Arts and Humanities	500	46	4.12	Japan	2.02	48.9	1.19	28.9	3.04
Chemistry	1,000	44	9.86	Singapore	5.33	54.0	3.55	36.0	1.82
Computer Science	2,000	44	4.52	Singapore	2.47	54.6	1.70	37.6	2.05
Dentistry	100	36	5.45	Hong Kong	2.25	41.2	2.06	37.8	3.89
Engineering	2,000	52	5.78	Hong Kong	2.97	51.3	2.32	41.1	3.08
Medicine	2,000	50	5.94	Belgium	4.12	69.3	2.92	49.2	1.40
Neuroscience	250	42	6.60	Sweden	4.54	68.8	3.65	55.3	2.38
Physics and Astronomy	1,000	55	7.53	Ireland	4.25	56.5	4.09	54.3	2.91
Social Sciences	1,000	48	3.06	Netherlands	1.96	64.0	0.78	25.5	0.21

## Discussion

This article described two new metrics for cross-country evaluation of scientific productivity that, while still using the widely available “number of papers - number of citations” data, improve over the much used CPP and h-index metrics. The metrics presented here are simple, objective and consistent along time, and their use would allow for meaningful and informative comparisons of the performance of countries. As an example, a remarkable improvement in Chilean relative performance could be detected here, and, on the other hand, the decrease in the United States and Russian performances could also be seen in a visually simple and direct way.

In the field of Scientometrics, measurement of impact in specific subject areas is influenced by frequency of publication, length of reference lists, and number of co-authors (Elsevier, 2018). Medicine, for instance, has a high frequency of publications in comparison with other 26 subject areas, according to the Scopus All Science Journal Classification. Moreover, nation publication patterns are highly heterogeneous both in their numbers, area, and impact, and, therefore, since the number of scientific publications has markedly increased in the last decades, comparisons with the world impact average (although common) may not represent the real change in a country’s scientific output. In addition, the world impact average also changes substantially within fields, relatively to the benchmark country. For example, for 2018, its value is 69% in Medicine 54% in Chemistry, 51% in Engineering, and 41% in Dentistry (Table 1).

Although essentially reporting the same information, the Relative CPP and the IRRS allow for different views of country performance in a dataset. Thus, IRRS indicates how countries “move” in a rank-list while the Relative CPP shows how much is needed for a country to get to “first place” – that is, how policy decisions are impacting their science sectors. Therefore, the ranking procedure (IRRS) is more sensitive to small changes, while the Relative CPP allows one to more globally visualize the extent of the difference between a country and the top ranker in the field.

The main limitation of the proposed indicators is the mentioned common problem among cross-country impact performance metrics: Relative performance evaluation can change contingent on a specific set of countries analyzed, and the inclusion of small-impact countries can greatly distort an analysis.

In summary, we presented here two metrics for cross-country comparison of scientific performance: The Relative CPP and the IRRS, and we hope that these will allow for a more informative discussion of government policies and their impact over the world scientific output.

## Author Contributions

RA, LB, DM, and MH contributed to the study design, data analysis, and manuscript drafting.

## Funding

Part of this research was funded by a Ministry of Education/CAPES grant (Code: 001), to whom we thank.

## Conflict of Interest

The authors declare that the research was conducted in the absence of any commercial or financial relationships that could be construed as a potential conflict of interest.
